# Budget Impact of Long-Acting Insulin Analogues: The Case in Brazil

**DOI:** 10.1371/journal.pone.0167039

**Published:** 2016-12-01

**Authors:** Fernanda O Laranjeira, Everton Nunes da Silva, Maurício G Pereira

**Affiliations:** University of Brasilia, Brasilia, Distrito Federal, Brazil; University of Colorado Denver School of Medicine, UNITED STATES

## Abstract

**Background:**

Long-acting insulin analogues for type 1 diabetes (T1D) treatment have been available on the Brazilian market since 2002. However, the population cannot access the analogues through the public health system.

**Objective:**

To estimate the incremental budget impact of long-acting insulin analogues coverage for T1D patients in the Brazilian public health system compared to NPH insulin.

**Methods:**

We performed a budget impact analysis of a five-year period. The eligible population was projected using epidemiological data from the International Diabetes Federation estimates for patients between 0–14 and 20–79 years old. The prevalence of T1D was estimated in children, and the same proportion was applied to the 15-19-year-old group due to a gap in epidemiological information. We considered 4,944 new cases per year and a 34.61/100,000 inhabitants mortality rate. Market share for long-acting insulin analogues was assumed as 20% in the first year, reaching 40% in the fifth year. The mean daily dose was taken from clinical trials. We calculated the bargaining power of the Ministry of Health by dividing the price paid for human insulin in the last purchase by the average regulated price. We performed univariate and multivariate sensitivity analyses.

**Results:**

The incremental budget impact of long-acting insulin analogues was US$ 28.6 million in the first year, and reached US$ 58.7 million in the fifth year. The total incremental budget impact was US$ 217.9 million over the five-year period. The sensitivity analysis showed that the percentage of T1D among diabetic adults and the insulin analogue price were the main factors that affected the budget impact.

**Conclusions:**

The cost of the first year of long-acting insulin analogue coverage would correspond to 0.03% of total public health expenditure. The main advantage of this study is that it identifies potential bargaining power because it features more realistic profiles of resource usage, once centralized purchasing is established as an economically sustainable strategy. Clinical guidelines restricting the use of insulin analogues would make the decision towards insulin analogue coverage more affordable.

## Introduction

The prevalence of diabetes is estimated to be 8.3% worldwide, affecting approximately 382 million people, of whom 5 to 10% have type 1 of the disease [1]. The last major pharmacological innovation for these patients took place approximately 20 years ago with the emergence of insulin analogues [2]. Although other insulins have been introduced over time (most recently the long-acting insulin analogues with flatter pharmacodynamic profile and the faster-acting insulin analogues for boluses), all pharmacological innovations for type 1 diabetes (T1D) continue in the same pattern as the analogues. Evidence suggests that insulin analogues show greater efficacy, cause fewer hypoglycemia episodes and promote a better quality of life than human insulin [3–5]. However, their higher cost has created barriers to accessing these technologies in health systems [6], particularly universal systems.

Insulin significantly contributes to the cost associated with diabetes, representing 24 [7] to 36% [8] of the treatment cost. The high cost has generated extensive discussion between parties favoring or opposing insulin analogues coverage [9,10]. In certain high-income countries, such as in Canada, the United Kingdom, Australia and France, individuals with T1D have access to insulin analogues [6,11,12], but under strict prescription criteria and only those in groups for which the analogues are cost-effective [13].

Brazil has the fourth largest number of diabetes patients worldwide, and type 1 affects approximately 0.31% of its population [1,14]. In a 2010 study, glycemic control was unsatisfactory in 87% of these patients (A1C > 7%) [15]. For T1D treatment, long-acting insulin analogues have been available on the Brazilian market since 2002. However, analogues are not accessible to the whole population through the national public health system. In 2014, the Brazilian Ministry of Health declined to provide rapid- and long-acting insulin analogues at the national level [16], based on two criteria: huge budget impact, due to high cost of insulin analogues, and the lack of evidence on efficacy.

The aim of this study is to estimate the incremental budget impact of long-acting insulin analogue coverage for T1D patients on the Brazilian public health system compared with current treatment provided by the public health system (NPH as basal insulin). This information may contribute to future decision-making processes not only in Brazil but also other developing countries that do not offer this type of insulin to their population.

## Materials and Methods

We carried out a budget impact analysis, in which we evaluated the affordability of offering long-acting insulin analogues (glargine or detemir) to T1D patients in the Brazilian public health system. A five-year time horizon was used disregarding conventional economic adjustments (discount rate and inflation) in accordance with recommendations from international methodological guidelines for budget impact analyses [17–19]. The calculations were performed using TreeAge^®^ Pro 2015 software.

It is worth noting that the treatment of T1D patients often combines two types of insulin: long-acting (basal profile) and rapid-acting (bolus profile). Although they are often used together by patients, international agencies have assessed these two types of insulin separately, for example: the National Institute for Health and Care Excellence (NICE) in the UK [20], the National Committee for Technology Incorporation (CONITEC) in Brazil [16], and the Canadian Agency for Drugs and Technologies in Health (CADTH) in Canada [21,22]. On this basis, we only included long-acting insulin in this study.

### Scenarios

The reference scenario was availability of NPH insulin only (currently available in public health system) as basal insulin for T1D patients. The alternative scenario was coverage of long-acting insulin analogues, glargine or detemir, to partially replace the NPH human insulin. We assumed that the insulin analogues exhibit similar efficacy and safety [3,4]. We did not include the insulin analogue degludec in the analysis because we have not identified any studies which compared degludec with NPH insulin.

### Target population

The target population was calculated using epidemiological studies. Our starting point was the International Diabetes Federation (IDF) estimate of 11.6 million diabetic people aged between 20 and 79 years in Brazil [1] and we used 5% as the percentage of T1D among adults with diabetes [14]. We included 31,100 diabetic people between 0 and 14 years old [14] (IDF estimate for children) in the model. Additionally, the prevalence was calculated for the 0-14-year-old age group by dividing the total number of T1D patients by the total 0-14-year-old population based on information from the Brazilian Institute of Geography and Statistics [23]. We reached a 0.654/1000 child prevalence. Subsequently, the same estimated prevalence was applied to the 15-19-year-old age group by multiplying the prevalence by the total 15-19-year-old population [23]. The target population was the total number of people estimated with T1D, which was obtained by summing the prevalence in the 0-14-year-old, 15-19-year-old and 20-79-year-old age groups.

We assumed 4,944 new cases per year [14] in the child age group to calculate the target population growth from the second year of the analysis onward. For this same period of analysis, we also used a T1D mortality rate of 34.61/100,000 inhabitants [24], calculated from Brazilian historical series published in 2012, which only included the T1D adult population.

### Costs

We considered direct costs associated with purchasing insulin over five years from the Brazilian public health system perspective.

Among the insulins analysed in this study, the Brazilian Ministry of Health only purchases NPH insulin, for which it has centralized purchasing. The Official Gazette, the official media vehicle of the Brazilian government, was used as the source for the values and quantities of the Ministry of Health’s most recent 10ml vials purchase [25].

To estimate the price that the Ministry of Health would pay to acquire long-acting insulin analogues (glargine and detemir), we simulated centralized purchasing (single buyer) gains based on the pattern verified with NPH. Thus, the ratio of the value paid by the Ministry of Health (centralized purchasing) and the regulated value (maximum selling price to the government [26]) was calculated. We calculated a 0.25 ratio; i.e., the value paid for NPH was 25% of the regulated value, representing a government bargaining power of 75%. We assumed that, for long-acting insulin analogues, the Ministry of Health would have 50% of the NPH bargaining power; i.e., the acquired value would be 62.5% of the regulated glargine and detemir values. For the sensitivity analysis, the lower limit was 100% of the bargaining power, and the upper limit was the regulated price (0% of the bargaining power).

To calculate the mean daily insulin dose, we used the mean dose from clinical trials that compared long-acting insulin analogues with NPH in children and adults. We used studies with more participants and adequate information on the mean dose. The insulin price was calculated per ml, and, at the end of the formula, the units were adjusted from IU to ml, and the mean dose was adjusted from daily to annual.

We used the mean daily dose from Home et al. [27] for adults over 19 years old and from Schober et al. [28] for children and adolescents under 19 years old (Table 1). Information on variations in value for both studies was poor; the amplitude was obtained using the minimum and maximum values and the difference between the baseline and end of the study. Thus, we used a 0.20 [29] standard deviation as the variation for all individuals.

**Table 1:** Average daily dose used per age group.

Age groups	Dose IU/day			
	NPH	SD	Analogue	SD
**Children**	21.10	±0.20	18.20	±0.20
**Adolescents**	21.10	±0.20	18.20	±0.20
**Adults**	21.00	±0.20	20.00	±0.20

IU: international units. NPH: Neutral Protamine Hagedorn insulin. SD: standard deviation.

The value derived from the last Ministry of Health NPH purchase was US$ 0.22 per ml [25]. The long-acting insulin analogue price was US$ 3.40 per ml. Thus, based on the mean daily dose per age group, we calculated a total annual cost of US$ 17.42 per child or adolescent who uses NPH insulin and US$ 225.64 per child or adolescent who uses a long-acting insulin analogue. For adults, the annual values were US$ 16.84 for NPH and US$ 247.96 for analogues. We used the following currency exchange rate: US$ 1.00 = R$ 4.00.

### Diffusion rate

We assumed a market share of 20% for long-acting insulin analogues in the first year, which increased 5 percentage points in the following periods until reaching 40% in the fifth year. This assumption was based on published and unpublished papers [30,31] which described utilization rates of long-acting insulin analogues between 14% and 25%.

### Sensitivity analysis

We performed univariate and multivariate sensitivity analyses through a tornado diagram, using confidence intervals, standard deviation or other types of measure of variation. The variation in parameters used for the sensitivity analysis was as follows.

- Percentage of T1D among total adult population with diabetes: 3 to 10%
- T1D mortality rate in adults aged 19 years and over: 16.68–54.38/100,000 adults [24]
- Incidence in the population up to 14 years old: 8.76–18.49 per 100,000 children [32]
- Mean dose: ± 0.20 IU per day [29]
- NPH insulin price: US$ 0.15–0.29 per ml
- Insulin analogues price: US$ 1.36–5.43 per ml
- Market share:
  - Year 1: 10%– 50%
  - Year 2: 15%– 60%
  - Year 3: 20%– 70%
  - Year 4: 25%– 80%
  - Year 5: 30%– 80%

## Results

### Target population

**Fig 1:**
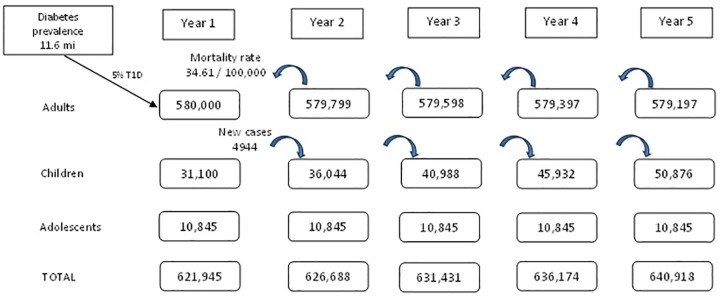
Model population dynamics for the budget impact analysis.

Fig 1 shows the dynamic population model assumed in our study, which projected the new cases, prevalence and mortality related to T1D. In the first year, we estimated 621,945 individuals had T1D, mainly adults (93%), followed by 6% children and 1% adolescents.

### Incremental budget impact analysis

The cost of the reference scenario (100% NPH insulin) was US$ 10.49 million in 2015, while the cost of the alternative scenario (80% NPH + 20% insulin analogues) was US$ 39.08 million in the same period, which results in an incremental budget impact of US$ 28.59 million. As long as the proportion of insulin analogues increases over the course of the time horizon in the alternative scenario, the incremental budget gets bigger. For example, the incremental cost was US$ 58.75 million in 2019. For the five-year period-analysis, the total incremental budget impact was US$ 217,860,488.31 (Table 2).

**Table 2:** Budget impact by scenario and incremental cost by year and for 5 years period (in US$ 1,000).

**Year 1–2015**			
**Scenario**	**Market share**	**Cost**	**Incremental cost**
Alternative	80% NPH + 20% analogue	39,079	28,588
Reference	100% NPH	10,491	-
**Year 2–2016**			
**Scenario**	**Market share**	**Cost**	**Incremental cost**
Alternative	75% NPH + 25% analogue	46,553	35,981
Reference	100% NPH	10,572	-
**Year 3–2017**			
**Scenario**	**Market share**	**Cost**	**Incremental cost**
Alternative	70% NPH + 30% analogue	54,125	43,473
Reference	100% NPH	10,652	-
**Year 4–2018**			
**Scenario**	**Market share**	**Cost**	**Incremental cost**
Alternative	65% NPH + 35% analogue	61,797	51,065
Reference	100% NPH	10,732	-
**Year 5–2019**			
**Scenario**	**Market share**	**Cost**	**Incremental cost**
Alternative	60% NPH + 40% analogue	69,567	58,754
Reference	100% NPH	10,813	-
**TOTAL**			
**Scenario**		**Cost**	**Incremental cost**
Alternative		271,12	217,86
Reference		53,26	-

### Sensitivity analysis

**Fig 2:**
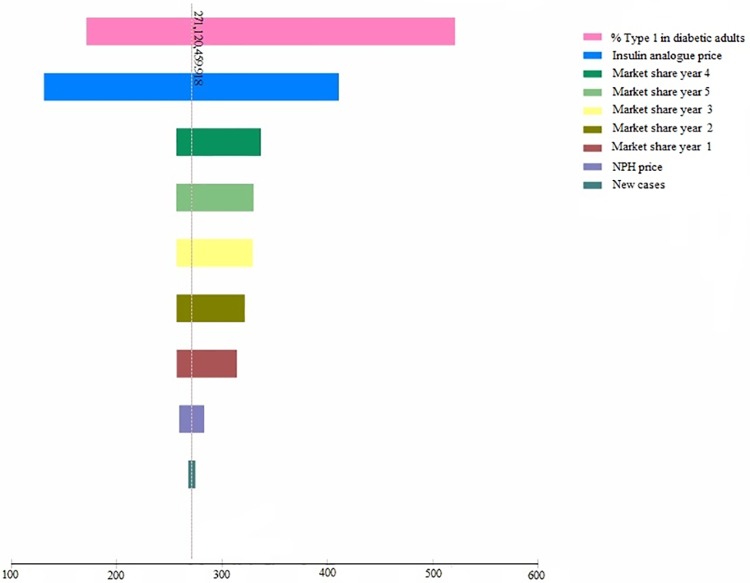
Variation of incremental budget impact according to changes on the assumptions of the model.

Based on the tornado diagram results, the two variables that most affected the budget were the percentage of T1D in the total adult diabetic population and the insulin analogue price, representing 54.19% and 34.68% of the total uncertainty, respectively. Market share played a small role on the sensitivity analysis, representing between 1.45% (Year 1) and 2.85% (Year 4) of total uncertainty. The remaining variables marginally affected the uncertainty (i.e., they had limited influence on the incremental budget impact) (Fig 2).

**Fig 3:**
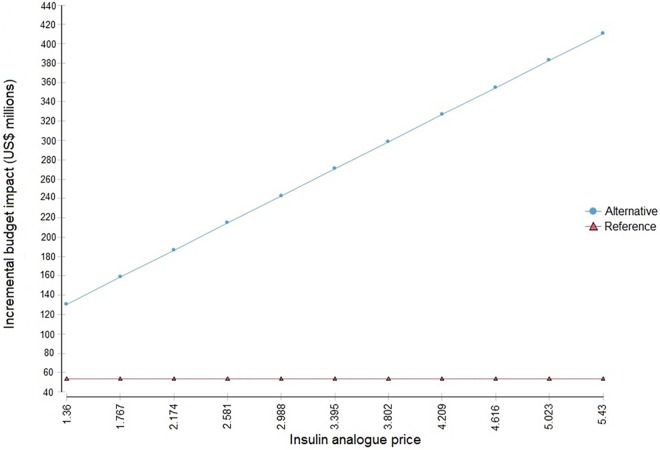
Budget impact evolution due to variation of insulin analogues price.

We ran a univariate sensitivity analysis on the two parameters that had the largest impact on the incremental analysis. For the long-acting insulin analogue price, at the lower limit, the Brazilian Ministry of Health would obtain the same discount for insulin analogues as currently used for NPH insulin. Under this assumption, the incremental budget impact would substantially decrease by more than 40% (US$ 217.8 million vs. US$ 130.8 million) (Fig 3).

The percentage of T1D among diabetic adults affected both scenarios but exerted a greater effect on the alternative one. Using 3% of T1Din total diabetic adults, the incremental budget impact decreases from US$ 217.8 million to US$ 171.2 million in five years. However, by using 10% of T1D in diabetic adults, the incremental impact reaches US$ 521.1 million over the same period.

## Discussion

Our study brings new evidence to support decision-making on the reimbursement of long-acting insulin analogues glargine and detemir for the Brazilian public health system, taking into consideration the bargaining power of centralized purchases at the national level. The results show an increment of US$ 217.8 million in the public budget over five years. For the first year of providing long-acting insulin analogues, the cost would correspond to 0.05% of the total Brazilian public health expenditure [33]. More specifically, considering the 2015 budget for the Specialized Component of the Pharmaceutical Assistance [34] (US$ 1.2 billion), in the first year, long-acting insulin analogues would represent 2.51% of the budget; in the fifth year, this percentage would increase to 5.8%.

In 2014, the Brazilian Ministry of Health carried out a budget impact analysis of introducing insulin analogues into the public health system, which estimated an impact of US$ 620 million [16], which is 2.84 times greater than our estimate. The difference arises from methodological differences, particularly for the mean dose (which was greater than in our analysis), insulin cost measurements (the price of long-acting insulin analogues was 38% greater than in our study) and the market share (the Brazilian Ministry of Health analysis included 100% of the target population).

Our study used the mean daily dose from clinical trials [27,28], which varied by age group. Observational studies, including Brazilian studies [7,15,35], found different doses among the population. However, we used information from studies with better methodological quality.

Our study is innovative because we introduced bargaining power into the model. The Brazilian Ministry of Health has used the centralized purchasing modality since 2009 [36,37]. Centralized purchasing increases the scale and provides a greater margin for price negotiation [38]. For NPH human insulin, the maximum regulated price was reduced by 75%. For the baseline, we adopted a conservative premise and assumed that, upon introducing long-acting insulin analogues into the system, the Brazilian Ministry of Health would receive a 37.5% discount, which is half of its bargaining power for NPH. Certain studies indicate that centralized purchasing reduces the price by, on average, 30 to 50% of the decentralized purchasing value [39–41]. Another study shows a need to decrease the price of analogues so that they become more attractive to the health system [42]. Therefore, it is important that insulin analogues are purchased nationally for better bargaining power compared with local negotiations.

For the market share, the need of the Brazilian T1D population for insulin analogues is unknown. Due to the lack of diffusion statistics, we used a conservative market entry profile, which peaked at 40% of the target population in five years. Aggressive market shares, such as 100% of the target population, would not be considered due to rational indication restrictions proposed by national and international guidelines [6,13,43,44,45]. These indication restrictions follow a global trend concerning similar restrictions proposed by other countries with public health systems.

Notably, Poland provides long-acting insulin analogues, but only for patients with severe hypoglycemia episodes. The coverage was the result of a therapeutic program based on the treatment’s success at decreasing hypoglycemia episodes after six months. In Poland, the success rate was 25% among T1D patients [31]; thus, the coverage was affordable. If the same access strategy were applied in Brazil, then the incremental budget impact would substantially decrease.

In Brazil, guidelines from the Ministry of Health have been restricting indications to treatment. There are some successful examples of the strategy, such as the rheumatoid arthritis, for which biological drugs are only provided for patients considered to be at a serious stage of the condition and who have been tested for all other disease-modifying-antirheumatic-drugs (DMARDs) [46]. Another example is multiple sclerosis, a disease for which the guideline is divided into three treatment lines [47]. Several kinds of cancer have clinical guidelines also structured in treatment lines that can limit the use of high-cost drugs to those in the population who would benefit most from this type of drug [48].

Although the public health system does not provide analogue insulin at the national level, there are some Brazilian states that already provide it at the local level. In these states, there are clinical guidelines that define the inclusion criteria for insulin analogue use, which are people: i) who have previously-diagnosed T1D; ii) have persistent bad glycemic control, after use of multiple daily injections with human insulin, documented by A1C tests (3 in the last 12 months) plus a clinician evaluation, detailing previous treatment algorithms with dosage and type of insulin used; iii) who have severe hypoglycemia (< 50mg/dl), regarding 2 or 3 episodes proven by laboratory tests and/or emergency reports on two different occasions, and iv) unawareness hypoglycemia. In these guidelines, bad glycemic control is defined as: A1C with 2 points above the superior limit of test [49].

It is worth noting that as we did not find Brazilian evidence that insulin analogues reduce the need for health resources, such as hospitalizations or complications, compared with NPH, we did not include them in our study. However, there is plenty of evidence on this issue in the international literature [50]. For example, evidence has shown that compared with NPH, insulin analogues decreases the risk of severe hypoglycemia episodes by 38% (OR 0.62, 95% CI 0.42–0.91) [4], the risk of nocturnal hypoglycemia episodes by 46% (p = 0.04) [51], the risk of hospitalization due to the first severe hypoglycemic event by 21.7% (95% CI 9.6–32.1%,p<0.001) [52], the risk of hypoglycemic coma recurrence by 36.3% (95% CI 8.9–55.5%, p = 0.014) [52], hospitalizations by 49% [53], the incidence of macrovascular complications by 48% [54], and annual costs [55].

Some limitations of our study should be acknowledged. First, we did not consider the indirect effect of insulin analogues on health services. According to the international literature, insulin analogues tend to decrease the need for health resources compared with NPH. Second, we only included long-acting insulin in our study, although T1D patients often used long-action insulin combined with rapid-acting insulin. A recent study carried out by the Brazilian Society of Diabetes estimated the budget impact of rapid-acting insulin analogues on the public health system would range between US$60.5 million and US$101 million in five years.

## Conclusion

In Brazil, states and municipalities have the autonomy to take decisions, including issues related to health technologies coverage. Thus, some Brazilian states already provide insulin analogues. However, based on our results, insulin analogue coverage should be part of a national policy because national price negotiation could achieve more affordable prices for the health system as a whole. In this context, clinical guidelines must effectively identify patients with a greater potential to benefit from insulin analogues with clear criteria for inclusion and maintenance in the program. Our analysis was conservative because we did not use the full potential of bargaining power and because we used a broad market share. New opportunities for research emerge from these results, notably on the prevalence of diabetes, the need for insulin analogues and use of real-world data for a new budget impact model.
